# The Effects of Integrating Visual Thinking Strategies on Intellectual Humility, Tolerance of Ambiguity, Empathy, and Observation Skills: A pre-post Study among Medical Students

**DOI:** 10.1007/s40670-026-02696-6

**Published:** 2026-03-13

**Authors:** Stefano Ardenghi, Marco Bani, Elena Silvia Sisca, Selena Russo, Giulia Rampoldi, Federico Zorzi, Alexia Del Greco, Maria Grazia Strepparava

**Affiliations:** 1 School of Medicine and Surgery, University of Milano-Bicocca, Via Cadore 48, Monza (MB), 20900 Italy; 2 Fondazione IRCCS San Gerardo dei Tintori, Monza, Italy; 3 Department of Biomedical Sciences, University of Sassari, Sassari, Italy; 4 Department of Earth and Environmental Sciences, University of Milano- Bicocca, Milano, Italy

**Keywords:** Medical education, Visual Thinking Strategies, Critical thinking, Empathy, Observation skills

## Abstract

**Background:**

Visual Thinking Strategies (VTS) are increasingly explored in medical education for their potential to foster key professional competencies, including intellectual humility, tolerance of ambiguity, empathy, and observational skills. However, empirical evidence supporting their educational impact remains limited.

**Methods:**

This pre-post controlled study with non-random group assignment based on administrative scheduling included 138 second-year Italian medical students (53.4% female; mean age = 20.20 ± 1.62) attending a mandatory communication skills module. The experimental group (*n* = 102) received a blended curriculum combining standard training with VTS-based sessions, while the control group (*n* = 36) followed the standard curriculum alone. Outcomes comprised three self-report questionnaires – the Comprehensive Intellectual Humility Scale (CIHS), the Multidimensional Attitudes Toward Ambiguity Scale (MATAS), and the Questionnaire of Cognitive and Affective Empathy (QCAE) – and two rubric-based measures of observational ability, administered only to the experimental group. Data were analyzed using 2 × 2 mixed-design ANOVAs (time [T0, T1] × group [experimental, control]) and repeated-measures t-tests.

**Results:**

Significant main effects of time emerged for CIHS subscales (Independence of intellect and ego, Respect for others’ viewpoints, Lack of intellectual overconfidence), Need for complexity and novelty of the MATAS, and Peripheral responsivity of the QCAE, indicating overall improvements across groups. No significant main effects of group or time × group interactions were observed. Conversely, rubric-based analyses revealed significant pre-post gains in observational and interpretive skills in the experimental group.

**Conclusions:**

VTS yielded effects on CIHS, MATAS, and QCAE comparable to the standard curriculum, while rubric-based findings suggest it enhances visual analysis and reflective observation, complementing traditional communication training.

## Introduction

Over the past two decades, medical education has increasingly embraced arts-based pedagogies as a means to cultivate essential clinical and interpersonal competencies that are both inadequately addressed in traditional curricula and challenging to teach through conventional didactic methods [1–3]. In response, a lot of educational programs have emerged globally through partnerships between medical schools and art institutions, signaling a growing recognition of the value of the arts in developing well-rounded clinicians [4]. One of the most promising methods in this domain is the Visual Thinking Strategies (VTS), a structured, discussion-based approach to art viewing originally developed for museum education [5]. Grounded in constructivist learning theory and informed by empirical research, VTS employs a facilitation protocol centered on three open-ended questions that promote close observation, evidence-based reasoning, and collaborative interpretation [6]. This approach has been adapted for use in medical education, where it has been employed to enhance competencies widely recognized as essential to effective and humanistic medical practice, particularly by fostering wonder and deeper engagement through the integration of arts and humanities into the curriculum [7].

By engaging students in facilitated discussions of ambiguous visual stimuli, VTS cultivates cognitive flexibility and intellectual humility, encourages hypothesis generation, and reinforces evidence-based reasoning – core features of critical thinking [8–10] and ambiguity tolerance [11]. The collaborative nature of the discussions, grounded in attentive listening and paraphrasing, has been shown to promote perspective-taking and empathic engagement [12]. Moreover, repeated exposure to complex artworks enhances learners’ capacity to attend to visual detail and integrate multiple visual elements, skills directly transferable to clinical observation [13, 14]. Within medical education, the development of these competencies is increasingly viewed as essential to preparing students for the complex demands of clinical practice [15, 16]. Such competencies are not accessory but central to physicians’ professional practice, as they shape how clinicians interpret information, engage with uncertainty, and relate to patients and colleagues.

Intellectual humility, understood as the capacity to recognize the limits of one’s knowledge and remain open to alternative interpretations [17], is increasingly recognized as a essential disposition for evidence-based reasoning. It supports reflective judgment, mitigates premature diagnostic closure, and fosters receptivity to new or conflicting evidence [18], enabling future physicians to engage in nuanced diagnostic reasoning, evaluate competing hypotheses, and make informed clinical decisions in complex and unpredictable scenarios [9]. Tolerance for ambiguity is equally vital, as clinical environments often involve incomplete data, shifting presentations, and the need to act despite diagnostic uncertainty [19]. Higher tolerance of ambiguity has been associated with greater psychological adaptability, reduced decisional stress, and more adaptive responses to clinical uncertainty [20], all of which are critical for sustainable clinical practice. Empathy extends beyond affective resonance to include the cognitive ability to understand others’ viewpoints, a competency essential for effective communication, shared decision-making, and patient-centered care [21]. It contributes not only to the quality of the patient-physician relationship, but also to improved adherence, satisfaction, clinical outcomes, and reduced work-related burnout [22]. Observational skills, in turn, form the foundation of physical examination and pattern recognition, and are crucial for the early detection of subtle clinical signs [23].

Systematic reviews of VTS [24, 25] and other visual art-based training initiatives [26] in medical education have highlighted their potential to address several of the profession’s pressing educational challenges, including the reduction of personal and work-related burnout [27]. However, while existing literature suggests positive outcomes, significant gaps remain regarding the design, implementation, and systematic evaluation of these interventions within medical curricula [28]. Further research is needed to clarify if and to what extent VTS contributes to specific educational outcomes and to assess its effectiveness in varied instructional contexts. The present study contributes to this effort by evaluating the effectiveness of an educational integration based on VTS for medical students. Drawing on the theoretical premises outlined above, we hypothesized that students who received the VTS-based educational intervention would exhibit higher levels of critical thinking, tolerance for ambiguity, empathy, and observational skills compared to those who followed the standard curriculum.

## Methods

### Procedure

This study adopted a pre-post design with a control group. It was conducted at the University of Milano-Bicocca, where the first two years of the 6-year medical program are preclinical, with practical clerkships beginning in the third year. Participants were recruited from second-year medical students who attended the mandatory practical sessions of the “Communication Techniques” module, part of the integrated course “Medicine and Society,” delivered during the first semester of the 2022/2023 academic year. Students were assigned to six training groups by the university’s administrative offices, which arranged the groups to ensure balanced sizes of approximately 20–30 students each. Group assignment was not random, but determined by the course timetable and institutional scheduling constraints. Two of these groups followed the standard training curriculum and served as the control group. Two different instructors facilitated these sessions. The remaining four groups formed the experimental group and were led by a single instructor trained in the application of VTS within medical education. Although different instructors were involved across groups, all sessions adhered to the same curricular framework and learning objectives. Instructors had comparable professional backgrounds as psychologists and psychotherapists, with experience in medical education. Before the start of the course, they participated in alignment meetings to agree on common teaching strategies and educational goals, ensuring consistency across sessions. All groups completed 12 h of training, delivered over three sessions of four hours each.

The standard curriculum included a structured sequence of activities: individual reflection on personal strengths and weaknesses in the communicative role of a future physician; small-group discussions of real-life or observed episodes of effective and ineffective communication between patients and healthcare providers; a collaborative drawing task in pairs, in which one student (the describer) conveyed an image verbally to another student (the drawer), who reproduced the image without visual access to the original; a theoretical introduction to core communication skills in healthcare; and role-playing exercises based on clinical scenarios, followed by reflective feedback sessions. The experimental group followed a blended approach, in which each session consisted of 2 h of standard training and 2 h of VTS-based activities. In contrast, the control group received standard training only, with no exposure to VTS or visual artworks. During the VTS sessions, students participated in guided observation and collective discussion of visual artworks. These sessions were structured around three core questions that scaffolded the discussion: “What’s going on in this picture?” encouraged students to offer initial interpretative hypotheses; “What do you see that makes you say that?” prompted them to support their ideas with visual evidence; and “What more can we find?” invited ongoing exploration and peer exchange, fostering a reflective and collaborative learning environment.

Participation in the study was voluntary and did not entail any academic or financial benefits. Before completing the questionnaires, participants signed an informed consent and were provided with detailed information regarding the study’s objectives and procedures. The survey was administered online. Although the system required responses to all items to facilitate comprehensive data collection, students were free to withdraw at any time. Questionnaires were submitted at two time points: at the beginning of the first session (T0) and after the final session (T1) of the training cycle. The interval between T0 and T1 was approximately one month, corresponding to the period over which the 12-hour training was delivered across three sessions. Data collection was conducted by a researcher unaffiliated with students’ instruction, thereby minimizing the risk of evaluative bias. To maintain anonymity while enabling the matching of pre- and post-intervention responses, each participant generated a unique alphanumeric code known only to themselves. To allow students in the control group to benefit from the educational experience based on VTS, a brief VTS session was organized after the T1 assessment. This post-assessment activity enabled control group participants to become familiar with the methodology and appreciate the type of work undertaken by their peers in the experimental group. The session was deliberately scheduled following the completion of data collection to avoid contamination of the results. The study received approval from the Ethics Committee of the University of Milano-Bicocca (Protocol number: 793) and was conducted in accordance with the ethical standards outlined in the Declaration of Helsinki.

## Materials

### Self-Report Questionnaires

Data were collected using three standardized self-report questionnaires and two analytic rubrics adapted for self-assessment. Specifically, the following instruments were completed by both the experimental and control groups: the Comprehensive Intellectual Humility Scale [29] (CIHS), the Multidimensional Attitudes Toward Ambiguity Scale [30] (MATAS), and the Questionnaire of Cognitive and Affective Empathy [31, 32] (QCAE). In addition, two rating rubrics originally developed by Milkova et al. [33] and Ferrara et al. [34] were adapted for self-report use to evaluate competencies related to observational, descriptive, and inference abilities, and were administered exclusively to the experimental group. Because these rubric-based measures were specifically designed to assess behaviors elicited during VTS sessions, they were administered only within the experimental group and were therefore not suitable for between-group comparisons. Participants also completed a brief socio-demographic questionnaire, which collected information on age, biological sex, nationality, type of high school attended, and self-reported level of interest in the arts.

Intellectual humility was assessed using the Italian back-translated version of the CIHS. This self-report instrument consists of 22 items and captures four distinct dimensions of intellectual humility: Independence of intellect and ego, which reflects the ability to engage in intellectual disagreement without perceiving it as a personal attack; Openness to revising one’s viewpoint, which indicate a willingness to change one’s mind in light of new evidence; Respect for others’ viewpoints, which pertains to valuing and considering perspectives that differ from one’s own; and Lack of intellectual overconfidence, which denotes an awareness of one’s cognitive limitations and fallibility. Participants rated each item on a 5-point Likert scale ranging from 1 (strongly disagree) to 5 (strongly agree). Higher scores indicate greater levels of intellectual humility across the four dimensions. In this study, the CIHS has demonstrated good internal consistency across its subscales (Cronbach’s α > 0.74).

Attitudes toward ambiguous situations were assessed using the Italian validated version of the MATAS. This self-report instrument was developed to capture the multifaceted nature of individuals’ reactions to ambiguity across affective, cognitive, and motivational domains. The scale consists of 30 items, each rated on a 7-point Likert scale ranging from 1 (strongly disagree) to 7 (strongly agree). The MATAS comprises three subscales: Moral absolutism, which assesses a rigid, dichotomous approach to moral judgment; Discomfort with ambiguity, which captures affective unease in uncertain or complex social contexts; and Need for complexity and novelty, which reflects a motivational preference for complexity, diversity, and cognitive stimulation. In this study, the MATAS has demonstrated good internal consistency for each of the three subscales (Cronbach’s α > 0.78).

Empathy was assessed using the Italian validated version of the QCAE. The QCAE is a 31-item self-report instrument rated on a 4-point Likert scale (1 = “strongly disagree” to 4 = “strongly agree”). It is designed to measure two core components of empathy: cognitive empathy and affective empathy. The cognitive dimension is composed of two subscales: Perspective taking (the ability to adopt another person’s point of view), and Online simulation (the tendency to imagine others’ emotional states as if they were one’s own); the affective dimension includes Emotion contagion (the tendency to automatically share the emotions of others), Proximal responsivity (emotional responsiveness to socially salient stimuli), and Peripheral responsivity (the tendency to experience empathy for fictional characters in films or novels). Higher scores on the QCAE indicate greater levels of empathy across its five dimensions. In this study, the QCAE has demonstrated good internal consistency across its subscales (Cronbach’s α > 0.79).

To evaluate participants’ visual literacy and descriptive abilities, the study employed the Scoring Rubric for Assessment of Visual Analysis (SRAVA) developed by Milkova et al. [33]. This analytic rubric was originally based on the Visual Literacy Competency Standards for Higher Education issued by the Association of College and Research Libraries. It comprises seven distinct categories, each rated on a 3-point scale [1–3], where higher scores indicate greater proficiency in observing, describing, and interpreting visual stimuli. The categories are: Composition, which evaluates the ability to observe the arrangement of figures or objects, describe their spatial relationships, and use effective descriptive language; Space and setting, which assesses understanding of spatial structure, perspective, and the articulation of foreground, middle ground, and background; Scale and viewpoint, which measures awareness of size relationships and the viewer’s position in relation to the image, including any distortions; Color, which captures attention to hue, saturation, repetition, and symbolic or expressive use of color; Lines and shapes, which evaluates recognition of geometric or organic forms, outlines, and how shapes are constructed and function visually; Light and light source, which assesses understanding of lighting effects, directionality, shadows, and their contribution to realism or mood; and Main theme and interpretation, which measures the ability to identify the main subject or action, integrate visual elements meaningfully, and provide a justified interpretation based on visual evidence. Each score level is anchored to explicit performance descriptors.

To assess participants’ skills in visual analysis and interpretation, the study employed the VTSkill rubric [34], an analytic scoring instrument specifically developed to evaluate core outcomes targeted by VTS-based interventions. The rubric was adapted from two established sources: the Student Thinking Assessment Rubric developed by the Visual Thinking Strategies Organization and the Scoring Rubric for Assessment of Visual Analysis proposed by Milkova [33]. The VTSkill rubric comprises four main competence domains: Critical Thinking, which assesses the ability to evaluate image characteristics and provide reasoned interpretations; Observation and attention, which measures the extent of visual detail captured by the participant; Linguistic expression, which evaluates the clarity, complexity, and coherence of the participant’s written response; and Problem solving/Inference ability, which captures the participant’s capacity to integrate visual elements and infer relationships. Each competence is rated on a five-point scale from 0 to 4, with higher scores indicating greater proficiency. Each score corresponds to a narrative descriptor that defines the observable criteria associated with each level of performance.

An ad hoc single-item measure was developed to assess participants’ interest in the arts and non-scientific/non-medical literature and to explore potential associations between individual interest in the humanities and the measured outcomes. Participants were asked: “How interested are you in art and non-scientific/non-medical literature?”. Responses were recorded on a 5-point Likert scale ranging from 1 (“Not at all”) to 5 (“Very much”), with intermediate options labeled as “A little”, “Moderately”, and “Quite a lot”.

## Visual Stimuli and Artwork Selection

The visual component of the VTS-based intervention consisted of a curated selection of 12 artworks, presented across three training sessions (4 paintings for each session) (Table 1). Artworks were selected based on three core criteria: [1] moderate to low public familiarity [2], a high degree of semantic ambiguity, and [3] the presence of emotionally salient content. Artworks were presented sequentially throughout each training session: paintings with more explicit and accessible narratives were shown first, followed by increasingly complex and ambiguous images.

**Table 1:** Overview of artworks presented during the training sessions

Training session	Title	Author	Year	Location
1	Painter’s family	Vladimir Makovsky	1893	National Art Museum of Azerbaijan
	San Camillo de Lellis salva gli ammalati dell’Ospedale San Spirito durante l’inondazione del Tevere del 1598	Pierre Hubert Subleyras	1746	Museo di Roma, Italy
	Waiting room II	George Tooker	1982	Vermont Arts Council, USA
	The rich soil down there	Kara Walker	2002	Museum of Fine Arts, Boston (MA), USA
2	The doctor	Luke Fildes	1891	Tate Gallery, London (UK)
	Crisi d’identità	Teresa Palombini	N.A.	PitturiAmo online gallery
	Trente-six expressions de têtes	Louis-Léopold Boilly	1822	Musée des Beaux-Arts, France
	Somewhere along the way (Part II)	Margo Kren	1997	Beach Museum of Art, Kansas State University, USA
3	The country doctor	Morgan Weistling	2013	Autry Museum of the American West, Los Angeles, USA
	Death in the sickroom	Edvard Munch	1895	Munch Museum, Oslo, Norway
	The two Fridas	Frida Kahlo	1939	Instituto Nacional de Bellas Artes, Mexico
	Le surréaliste	Victor Brauner	1947	Musée Unterlinden, Colmar, Alsazia

*Notes*. *N.A*.Not available

## Statistical Analyses

Before conducting inferential analyses, descriptive statistics were computed for each outcome measure, including means and standard deviations (SD). Skewness and kurtosis coefficients were used to assess the distributional characteristics of the variables and the assumption of normality, where relevant. Independence of observations was ensured by the study design. Homogeneity of variances for the between-subjects factor was assessed using Levene’s test and was met for all analyses. For repeated-measures factors (time: T0 *versus* T1), the assumption of sphericity was inherently satisfied given the two-level design. First, analyses comparing the two control groups revealed no significant differences at T0 or T1 across socio-demographics and outcome measures; therefore, the two groups were combined into a single control group for subsequent analyses. Second, analyses were performed to examine T0 differences between the experimental and control groups and among the training groups in terms of socio-demographic variables (e.g., biological sex, age, nationality, type of high school attended, and level of interest in the arts), and pre-intervention scores on CIHS, MATAS, and QCAE. Independent samples t-tests and analysis of variance were used for continuous variables, and chi-square tests were applied for categorical variables. A series of 2 × 2 mixed-design ANOVAs (time [T0, T1] × group [experimental, control]) were conducted to examine whether pre-post changes differed significantly between groups for the CIHS, MATAS, and QCAE scales. Partial eta-squared (η²p) was reported as a measure of effect size for these analyses. In addition, to further evaluate the effectiveness of the VTS-based educational intervention, repeated-measures t-tests were performed on SRAVA and VTSkill rubric dimensions for the experimental group only. Cohen’s d was calculated for each analysis to quantify the magnitude of observed differences. All tests were two-tailed, and statistical significance was set at *p* < 0.05. All statistical analyses were conducted using IBM SPSS Statistics (version 29.0.2.0).

## Results

A total of 141 second-year medical students were invited to participate in the study; 138 consented and completed the assessments, yielding a response rate of 97.9%. Of these, 102 (73.9%) were assigned to the experimental group and 36 (26.1%) to the control group. Descriptive characteristics of the study sample are presented in Table 2. The sample consisted of Italian students, with a mean age of 20.20 **±** 1.62 years. Skewness and kurtosis values for all measures fell within the acceptable range, indicating that the data were approximately normally distributed and thus appropriate for parametric analyses. No statistically significant differences were found between the experimental and control groups at T0 on any outcome measures, including the CIHS, MATAS, and QCAE. Likewise, no statistically significant differences emerged between the groups in the distribution of biological sex, age, nationality, type of high school attended, or level of interest in the arts. Furthermore, no significant differences were observed among students assigned to different training groups. In addition, no statistically significant associations were observed between socio-demographic variables and any baseline outcome measures. These preliminary analyses confirmed a homogeneous distribution of variables across all training groups and indicated that background characteristics did not confound participants’ baseline scores on the CIHS, MATAS, QCAE, or the two rubrics assessing observational, descriptive, and inferential skills.

**Table 2:** Sample characteristics

	Total (*n* = 138)	Experimental group (*n* = 102)	Control group (*n* = 36)	
	*N* (%)	*N* (%)	*N* (%)	*p*
Biological sex				0.454
Female	74 (53.4)	55 (53.9)	19 (52.8)	
Male	64 (46.6)	47 (46.1)	17 (47.2)	
High school				0.576
Scientific high school	102 (73.6)	75 (73.5)	27 (75.0)	
Classical high school	27 (19.8)	20 (19.6)	7 (19.4)	
Technical institute	2 (1.5)	1 (1.0)	1 (2.8)	
Vocational institute	0 (0.0)	0 (0.0)	0 (0.0)	
Other	7 (5.1)	6 (5.9)	1 (2.8)	
Interest in the arts				0.650
Not at all	4 (2.6)	3 (2.9)	1 (2.8)	
A little	21 (14.9)	16 (15.7)	5 (13.9)	
Moderately	46 (33.3)	34 (33.3)	12 (33.3)	
Quite a lot	50 (36.0)	36 (35.3)	14 (38.9)	
Very much	18 (13.2)	13 (12.8)	5 (13.9)	

Significant main effects of time were found only for the CIHS subscales Independence of intellect and ego [F(1, 136) = 19.88, *p* < 0.001, η²*p* = 0.13], Respect for others’ viewpoints [F(1, 136) = 5.02, *p* = 0.026, η²*p* = 0.04], and Lack of intellectual overconfidence [F(1, 136) = 23.30, *p* < 0.001, η²*p* = 0.15], as well as for Need for complexity and novelty of the MATAS [F(1, 136) = 4.90, *p* = 0.028, η²*p* = 0.04] and Peripheral responsivity of the QCAE [F(1, 136) = 5.40, *p* = 0.021, η²*p* = 0.04], all indicating significant improvements from pre- to post-test across both groups. For all scales, neither the main effect of group nor the time × group interaction reached statistical significance, indicating that no differential effects between training conditions were detectable under the present study conditions (Table 3).

**Table 3:** Pre-post differences for CIHS, MATAS, and QCAE: results of 2 × 2 mixed-design ANOVAs (time × group)

Variable	Group	T0	T1	
		Mean ± SD	Mean ± SD	*p*
CIHS				
Independence of intellect and ego	Total	3.27 ± 0.77	3.90 ± 0.93	< 0.001
	E	3.34 ± 0.71	4.03 ± 0.93	
	C	3.19 ± 0.82	3.76 ± 0.92	
Openness to revising one’s viewpoint	Total	4.15 ± 0.75	4.38 ± 0.47	0.321
	E	4.17 ± 0.74	4.35 ± 0.54	
	C	4.12 ± 0.76	4.41 ± 0.40	
Respect for others viewpoints	Total	4.20 ± 0.75	4.41 ± 0.42	0.026
	E	4.22 ± 0.65	4.39 ± 0.42	
	C	4.17 ± 0.85	4.43 ± 0.42	
Lack of intellectual overconfidence	Total	3.17 ± 0.55	3.46 ± 0.67	< 0.001
	E	3.21 ± 0.59	3.53 ± 0.62	
	C	3.12 ± 0.51	3.38 ± 0.72	
MATAS				
Moral absolutism	Total	3.29 ± 1.08	2.99 ± 1.08	0.553
	E	3.13 ± 1.15	2.89 ± 0.98	
	C	3.44 ± 1.01	3.09 ± 1.17	
Discomfort with ambiguity	Total	4.68 ± 1.15	4.48 ± 1.24	0.502
	E	4.65 ± 1.12	4.43 ± 1.06	
	C	4.70 ± 1.17	4.53 ± 1.41	
Need for complexity and novelty	Total	4.34 ± 1.32	4.62 ± 1.06	0.028
	E	4.08 ± 1.51	4.56 ± 1.09	
	C	4.60 ± 1.13	4.68 ± 1.02	
QCAE				
Perspective taking	Total	2.83 ± 0.57	2.92 ± 0.55	0.404
	E	2.87 ± 0.49	2.92 ± 0.51	
	C	2.79 ± 0.65	2.91 ± 0.59	
Online simulation	Total	3.11 ± 0.54	3.14 ± 0.40	0.791
	E	3.12 ± 0.44	3.15 ± 0.34	
	C	3.09 ± 0.63	3.12 ± 0.45	
Emotion contagion	Total	2.55 ± 0.69	2.61 ± 0.70	0.453
	E	2.54 ± 0.60	2.63 ± 0.65	
	C	2.55 ± 0.77	2.59 ± 0.75	
Proximal responsivity	Total	2.92 ± 0.58	2.86 ± 0.65	0.351
	E	2.91 ± 0.53	2.91 ± 0.57	
	C	2.93 ± 0.62	2.81 ± 0.73	
Peripheral responsivity	Total	2.50 ± 0.59	2.68 ± 0.60	0.021
	E	2.53 ± 0.54	2.70 ± 0.62	
	C	2.47 ± 0.64	2.65 ± 0.58	

Notes. *E*  Experimental group, *C*  Control group

For the SRAVA dimensions, significant pre-post improvements emerged in Composition [t(101) = -3.09, *p* = 0.003, d = 0.51], Scale and viewpoint [t(101) = -2.46, *p* = 0.017, d = 0.31], Colors [t(101) = -2.28, *p* = 0.026, d = 0.29], Lines and shapes [t(101) = -2.89, *p* = 0.005, d = 0.41], and Main theme and interpretation [t(101) = -2.12, *p* = 0.038, d = 0.29]; **as** for the VTSkill Rubric, a significant pre-post improvement was found only for the Observation skill dimension [t(101) = -2.17, *p* = 0.034, d = 0.39] **(**Table 4**)**.

**Table 4:** Pre-post differences in SRAVA and VTSkill Rubric scores for the experimental group (*n* = 102): results of repeated-measures t-tests

Variable	T0	T1	
	Mean ± SD	Mean ± SD	*p*
SRAVA			
Composition	2.63 ± 0.77	3.00 ± 0.68	0.003
Space and setting	2.91 ± 0.60	3.00 ± 0.53	0.113
Scale and viewpoint	2.65 ± 0.58	2.83 ± 0.58	0.017
Colors	2.72 ± 0.68	2.90 ± 0.54	0.026
Lines and shapes	2.51 ± 0.82	2.82 ± 0.69	0.005
Light and light source	2.94 ± 0.79	3.00 ± 0.69	0.418
Main theme and interpretation	3.01 ± 0.72	3.19 ± 0.52	0.038
VTSkill Rubric			
Critical thinking	3.16 ± 0.63	3.27 ± 0.68	0.094
Observation skill	2.69 ± 0.72	2.97 ± 0.68	0.034
Linguistic expression	3.09 ± 0.76	3.26 ± 0.65	0.188
Problem solving/Inference ability	3.13 ± 0.64	3.27 ± 0.58	0.233

## Discussion

The present study investigated the effectiveness of integrating VTS into a communication skills training module for second-year medical students, using a pre-post design with a control group and standardized self-report and rubric-based measures of intellectual humility, tolerance for ambiguity, empathy, and visual observational skills. Although both experimental and control groups showed statistically significant pre-post improvements across several outcomes, the observed effect sizes were small, suggesting modest incremental gains rather than substantial changes. No group-specific effects emerged under the study conditions potentially reflecting the brief duration of the intervention, the dispositional nature of the constructs assessed, and the exploratory nature of the study.

This study employed intellectual humility as a proxy measure of critical thinking due to the conceptual convergence between these constructs. As outlined in the literature [8], critical thinkers are characterized by their awareness of the limits of one’s own knowledge, a willingness to revise one’s viewpoints in light of new evidence, and openness to alternative perspectives. Significant pre-post improvements were found in the CIHS subscales Independence of intellect and ego, Respect for others’ viewpoints, and Lack of intellectual overconfidence across both groups, indicating that the communication skills module – regardless of the inclusion of VTS – supported the development of a more open, self-aware, and reflective intellectual stance. These findings are consistent with the notion of “staying open to possibilities” in medical education [35] and align with the theoretical underpinnings of VTS, which emphasize collaborative meaning-making, tolerance of interpretive plurality, and the suspension of premature judgment [36]. The absence of significant time × group interactions suggests that, under the present study conditions, the VTS-based intervention did not produce differential effects on intellectual humility. This may be because the standard curriculum already includes pedagogical elements that promote the exploration of multiple interpretations of interpersonal situations and constructive peer dialogue. This may have helped students distinguish intellectual disagreement from personal threat and engage more openly with differing viewpoints, as noted in previous studies [37].

Interestingly, a significant overall pre-post improvement was observed for the Need for complexity and novelty subscale of the MATAS, suggesting that both training conditions may have contributed to fostering a greater appreciation for complexity and new perspectives. Although this construct does not correspond to a specific clinical skill, it reflects a motivational orientation toward engaging with complexity rather than avoiding it, a disposition that is increasingly recognized as relevant for medical training [38]. In clinical practice, physicians are routinely required to navigate complex, ill-structured problems and integrate multiple, sometimes conflicting sources of information. From this perspective, greater tolerance for complexity and novelty may indirectly support adaptive clinical reasoning and learning in uncertain environments. Nevertheless, no significant group or time × group effects were found, suggesting that the VTS-based curriculum did not produce differential effects on tolerance of ambiguity that were detectable under the present study conditions. This partially aligns with the findings of Bentwich and Gilbey [11], who reported increased tolerance for multiple interpretations following a brief VTS session, although their conclusions were based on immediate self-reported impressions rather than standardized pre-post assessments with validated instruments. One possible explanation for the limited differential effects is the trait-like nature of ambiguity tolerance, which is generally considered resistant to short-term change [39–41]. While art-based interventions such as VTS can prompt reflection on uncertainty and interpretive plurality, more emotionally salient or prolonged experiences may be necessary to produce distinctive changes. In line with this perspective, Geller et al. [19] observed that tolerance for ambiguity in medical students evolved over the course of extended training, suggesting that shifts in this construct may emerge more clearly over longer timescales and within complex, real-world professional contexts.

A significant overall pre-post improvement was observed for Peripheral responsivity, a QCAE subscale reflecting the tendency to experience affective empathy toward fictional, imagined, or indirect stimuli [31, 32]. Although this dimension does not directly assess empathy in real clinical encounters, it captures an underlying dispositional openness to others’ emotional experiences, even when these are mediated or hypothetical. Such a capacity is increasingly considered relevant in medical education, as it may support perspective-taking, narrative understanding, and responsiveness to patients’ subjective experiences, particularly when emotional cues are subtle or indirectly expressed [42]. This enhancement, which emerged across both groups, suggests that no differential effects of the VTS-based intervention on this empathy dimension were detectable under the present study conditions. Rather, the observed pre-post change may reflect the broader educational impact of the communication module, which encourages students to reflect on emotionally nuanced situations, even when not directly experienced. The activities involving observation and discussion of interpersonal dynamics, whether through artworks or traditional training materials, likely encouraged reflection on emotional states and relational contexts, thereby fostering a more flexible and broader empathic capacity. It is important to acknowledge, however, that empathy is a multifaceted and developmentally complex competence [43, 44] that typically evolves over extended periods of time [45–47]. Moreover, its emergence and consolidation are shaped by a variety of dispositional factors, including personality traits [48], personal values [49], emotional intelligence [50], attachment styles [51], mindfulness facets [52], and emotion regulation strategies [53, 54]. Therefore, it is not surprising that a brief experiential intervention – such as the 12-hour training offered in this study – did not yield widespread changes across all dimensions of empathy.

Turning to the rubric-based assessments, our findings support the role of VTS in cultivating foundational visual and interpretive competencies in health professions education. Students in the experimental group demonstrated significant improvements in several dimensions of the SRAVA, including Composition, Scale and viewpoint, Colors, Lines and shapes, and Main theme and interpretation. These gains suggest that structured visual analysis exercises can enhance students’ capacity to process complex visual information, attend to formal and relational features, and articulate well-founded interpretations. These skills are central to clinical observation and diagnostic accuracy. Similarly, the Observation Skill domain of the VTSkill Rubric showed significant improvement, indicating that students exposed to VTS developed a greater ability to accurately attend to visual detail and describe scenes. These results align with previous studies that demonstrated the impact of VTS on strengthening observational acuity. For instance, Honan Pellico et al. [14] found that nursing students who actively observed artworks produced significantly more objective clinical findings and a broader range of differential diagnoses. Similarly, Agarwal et al. [26] showed that first-year medical students who underwent VTS training spent more time analyzing clinical images, used a greater number of descriptive terms, and generated more clinically relevant observations. In addition, Feen-Calligan et al. [13] observed enhanced awareness of observational skills and implicit bias following VTS sessions, suggesting broader benefits for diagnostic sensitivity and cultural competence. Finally, Ghorbani et al. [55] found that, in the short term, the VTS intervention group outperformed the control group in number of observations, descriptive vocabulary, and time spent analyzing images, while in the long term only the time spent describing clinical observations remained significantly higher compared to the control group. It should be noted that, although these rubrics were originally designed for external rating, they were adapted for student self-assessment in the present study. This modification may have reduced score objectivity and should be taken into account when interpreting the magnitude and meaning of the observed improvements.

## Strengths and Limitations

Several limitations should be considered when interpreting the results of this study. First, participants were not randomly assigned to experimental and control conditions. This may introduce selection bias, despite no significant baseline differences being detected between groups in demographic variables or pre-intervention scores. Second, the unequal group sizes may have affected variance stability and statistical power, even though assumptions of normality and homogeneity of variance were met. Third, different instructors were involved across groups, with the experimental condition facilitated by a single instructor trained in VTS and the control condition led by two instructors. Although all sessions adhered to the same curricular objectives, instructor-related effects cannot be fully ruled out and may have contributed to the observed outcomes. Fourth, the relatively short duration of the intervention may have constrained the depth and generalization of the learning outcomes, particularly in relation to more stable dispositional traits such as tolerance for ambiguity; additionally, the lack of follow-up assessments limits the ability to draw conclusions regarding the sustainability of the observed improvements beyond the immediate post-intervention period. Fifth, reliance on self-report instruments introduces the potential for social desirability bias or limited introspective accuracy, particularly in domains such as empathy and humility. Although rubric-based measures complemented the self-report data, these were adapted for self-assessment rather than applied by external raters and were administered only within the experimental group, limiting both their objectivity and the ability to determine whether observed improvements reflect VTS-specific effects or more general learning progression. Additionally, the study sample was drawn from a single medical school in Italy, which may limit the generalizability of the findings to other educational, cultural, and training contexts. Despite these limitations, the study also presents several notable strengths. The use of validated instruments and theory-driven constructs lends robustness to the evaluation of complex educational outcomes. Moreover, the intervention was implemented within a real curricular context rather than an artificial or extracurricular setting, enhancing its practical relevance for medical education. Finally, the inclusion of a control group receiving an active standard curriculum allows for a more meaningful interpretation of the added value of VTS-based training.

## Conclusions

This study provides preliminary evidence on the potential effectiveness of integrating VTS into a curricular communication skills module for pre-clinical medical students. While no differential effects between the VTS-based and standard curricula were observed for intellectual humility, tolerance for ambiguity, or empathy, both groups showed significant overall improvements across these domains, suggesting that the standard training itself effectively supports key educational outcomes. Within the VTS group, rubric-based analyses indicated improvements in observational and interpretive skills; however, in the absence of parallel rubric-based assessments in the control group, these findings should be interpreted as exploratory and descriptive rather than as definitive evidence of intervention-specific effects. Nonetheless, these results suggest that VTS may represent a promising complementary approach to traditional communication training, particularly in its potential to foster visual analysis and reflective observation abilities. Future research should replicate and extend these findings through randomized controlled trials, multi-institutional studies, and longitudinal follow-ups to assess the durability and transferability of these skills into clinical practice. In the meantime, these results encourage further curricular experimentation with structured visual art-based methodologies as tools to enrich the development of humanistic and observational competencies in future physicians.

## Data Availability

The datasets used and/or analysed during the current study are available from the corresponding author on reasonable request.
